# Gut microbiota, chemotherapy and the host: the influence of the gut microbiota on cancer treatment

**DOI:** 10.3332/ecancer.2018.868

**Published:** 2018-09-05

**Authors:** Anna Louise Pouncey, Alasdair James Scott, James Leslie Alexander, Julian Marchesi, James Kinross

**Affiliations:** Centre for Digestive and Gut Health, Department of Surgery and Cancer, Imperial College London, London SW7 2AZ, UK

**Keywords:** gut, microbiome, cancer, oncology, chemotherapy, pharmacobiomics

## Abstract

The gut microbiota exists in a dynamic balance between symbiosis and pathogenesis and can influence almost any aspect of host physiology. Growing evidence suggests that the gut microbiota not only plays a key role in carcinogenesis but also influences the efficacy and toxicity of anticancer therapy. The microbiota modulates the host response to chemotherapy via numerous mechanisms, including immunomodulation, xenometabolism and alteration of community structure. Furthermore, exploitation of the microbiota offers opportunities for the personalisation of chemotherapeutic regimens and the development of novel therapies. In this article, we explore the host-chemotherapeutic microbiota axis, from basic science to clinical research, and describe how it may change the face of cancer treatment.

## Introduction

The microbial commensals of the gastrointestinal system, the gut microbiota, outnumber the cells of the human body, and comprise the largest surface area of microbial interaction with the host immune system [1]. This dynamic interface, consisting of multiple metabolic, immunological and inflammatory pathways, exists in a delicate balance between symbiosis and pathogenesis and can influence many aspects of host physiology. While there is mounting evidence that the gut microbiome plays a key role in carcinogenesis [2], the advancement of high-throughput sequencing, metabolite profiling and bioinformatics algorithms is broadening our understanding of the interactions of the gut microbiota further still. In particular, a relatively novel area of research, ‘pharmacomicrobiomics’ (the study of the effect of microbiota on drug disposition, action and toxicity) is revealing the integral role that microbiota plays in the efficacy and toxicity of cancer treatment [3].

Pharmacomicrobiomics has the potential to enhance therapeutic efficacy and abrogate side effects by manipulating host-chemotherapeutic-microbiota interactions and for personalisation of chemotherapy regimens based on the evaluation of an individual’s microbiome (the genetic composition of their microbiota) [4]. Increased understanding of the complex interplay between gut microbiota and the immune system may also generate novel chemotherapeutic approaches [5, 6].

## Host-chemotherapeutic-microbiota interaction

Gut microbiota can modulate the host response to chemotherapy through numerous mechanisms, including immune interactions, xenometabolism and altered community structure [6]. Interaction with the immune system occurs both intra-luminally and within lymphoid organs following chemotherapy-induced bacterial translocation [7–9]. The microbiota directly metabolises chemotherapeutic medication and may produce toxic secondary metabolites. They also exert an indirect effect on host-chemotherapeutic metabolism through modification of the host microenvironment [10]. As the gut microbiota develops in synchrony and symbiosis with the host, its composition and functionality are highly individualised [11]. During the course of anticancer treatment, the community structure of gut microbiota is affected by multiple factors, such host environment and diet, surgical intervention, use of adjuvant medication (such as antibiotics) and the effect of the chemotherapy administered. Many of these factors generate dysbiosis, a perturbation of the microbial community, which disrupts the symbiotic relationship with the host. Immune interactions, xenometabolism and altered community structure can all cause adverse side effects, and impair chemotherapeutic outcome [12]. Analysis and manipulation of gut microbiota may, therefore, become a vital component for the development of personalised and effective anticancer therapy [6].

## Immunomodulation

Modification of the immune response by the microbiota plays a key role in determining chemotherapeutic response. At the mucosal surface, constant interaction between gut microbiota and the innate and adaptive immune systems have been shown to set the immune tone and regulate inflammation [5]. Chemotherapeutic medication can damage the mucosal epithelium, triggering bacterial translocation. While this may cause systemic infection, increased exposure to potential pathogens can also prime the adaptive immune system, augmenting chemotherapeutic response in the host [7–9]. The mechanisms by which immunomodulation can occur are diverse. Bacterial translocation and T-helper 17 cell activation enhance the action of cyclophosphamide; intraluminal myeloid cell activation enhances the action of oxaliplatin; and microbiota induced T-cell activation is a key in facilitating novel anticancer immunotherapy [9, 13].

The interaction among the immune system, microbiota and cyclophosphamide (CTX) (a commonly used alkylating agent), has been well characterised in both basic and clinical studies. Research by Viaud *et al* [9] using a murine sarcoma model showed that therapeutic response to CTX was greater in mice with healthy gut microbiota than in mice raised in a germ-free environment. Moreover, depletion of gram-positive gut bacteria with vancomycin pre-treatment resulted in a poor therapeutic response compared to untreated controls [9]. The authors elucidated that transmucosal translocation of specific bacteria (such as *Lactobacillus spp., Enterococcus hirae*) into mesenteric lymph nodes and the spleen, stimulated T-helper 17 (Th17) cell differentiation resulting in an antitumour adaptive immunological response. Fewer Th17 cells were observed in the spleens of vancomycin pre-treated mice and adoptive transfer of pathogenic Th17 cells from untreated mice could re-establish a therapeutic response [9, 14]. Further investigation demonstrated that administration of *E. hirae* in antibiotic pre-treated mice could also restore response to CTX [13]. These findings have been corroborated by clinical studies. Among patients with end-stage lung and ovarian cancer, a memory Th1 immune response towards *E. hirae* was found to be a positive predictor of progression-free survival [6, 10, 11, 14, 17].

The impact of microbiota and myeloid cell interaction on chemotherapeutic efficacy was investigated by Iida *et al* [8]*,* in a murine lymphoma model. Antibiotic pre-treatment of mice with subcutaneous EL4 lymphomas reduced both DNA damage by oxaliplatin (a DNA cross-linking agent) and the expression of genes responsible for reactive-oxygen species (ROS) generation by myeloid cells. *Cybb -/-* mice, deficient in NADPH oxidase, an enzyme critical for the production of ROS, also respond poorly to oxaliplatin. It was concluded that a healthy microbiota enhances the antitumour effect of oxaliplatin by priming of myeloid cells for ROS release, enhancing inflammatory cytokine production, and thus tumour eradication [8]. A similar effect was observed with CpG oligodeoxynucleotides (CpG OGN). CpG OGN are synthetic mimics of microbial DNA, which bind to TLR9, and can be injected to stimulate a tumour-eradicating immunological response in mice. In germ-free and antibiotic-treated mice, myeloid cell inflammatory cytokine production and the anticancer adaptive immune response to intra-tumoural administration of CpG OGN were reduced. This could be restored by inoculation with *Alistipes shahii* [8].

Immune checkpoint inhibitors, which target T-cell regulatory pathways, display promising results in patients with lung cancer or advanced melanoma [15]. It was demonstrated that treatment with a synthetic monoclonal antibody against cytotoxic T-lymphocyte-associated protein 4 (CTLA-4, ipilimumab) could control established MCA205 sarcomas in mice with healthy gut bacteria. However, germ-free or antibiotic-treated mice displayed no response to the CTLA-4 blockade. Melanoma patients responsive to CTLA-4 display T cell reactions specific for *Bacteroides* species; therapeutic reaction to ipilimumab in germ-free mice could be salvaged with the administration of *Bacteriodes fragilis* or adoptive transfer *B. fragilis*-specific T cells. This indicates that the interaction between *Bacteriodes* species and the immune system plays a significant role in enabling the CTLA-4 induced antitumour response [4].

In another study, Sivan *et al* [16] observed differential melanoma growth in genetically similar mice sourced from two suppliers—Taconic Farms (Tac) and Jackson Laboratory (Jax). Tumours grew more aggressively in Tac mice compared to Jax mice and this effect could be abrogated by either co-housing or by faecal transfer from Jax mice. Faecal transfer in the opposite direction did not confer increased tumour growth. Furthermore, chemotherapeutic response to antiprogrammed cell death 1 ligand-1 monoclonal antibodies (anti-PD-L1, pembrolizumab) was impaired in Tac mice compared to Jax mice. The authors determined that the attenuated basal tumour growth and enhanced anticancer response to anti-PD-L1 seen in Jax mice were due to the presence of *Bifidobacterium spp* in their gut flora which are believed to interact with dendritic cells to activate T-cells and stimulate a protective anticancer response. Administration of *Bifidobacterium spp.* to Tac mice was able to slow basal tumour growth to enhance the antitumour effect of anti-PD-L1 [16].

## Metabolism

The gut microbiota has the potential to directly metabolise chemotherapeutic drugs and also to modify the host metabolic milieu, indirectly altering host-chemotherapeutic metabolism [17, 18]. Recent research using the *Caenorhabditis elegans* nematode worm (*C. elegans*) as a model for symbiotic host-microbial interactions suggests that the variable clinical response to the first-line chemotherapy for colorectal cancer, fluoropyrimidines, may be due, not only to genetic polymorphisms but also to variations in gut microbiota [10, 19]. Investigations led by Garcia-Gonzalez *et al* [19] tested the efficacy of camptothecin (CPT), 5-fluorouracil (5-FU) and 5-fluoro-2’-deoxyuridine (FUDR) in *C. elegans,* which were fed with either *E. coli* or *Comamonas* bacteria. As fluoropyrimidines inhibit cell division and impair nematode fertility, the dose of chemotherapeutic needed to prevent live progeny could be assessed. It was found that bacterial species affect the response to chemotherapeutics differently. Nematodes fed with *E. coli* were two orders of magnitude more sensitive to the sterilising effect of FUDR. Genetic screening and the restorative effect of uracil supplementation were used to elucidate that ribonucleic acid (RNA) metabolism (conversion of 5-FU and FUDR to fluorouridine monophosphate) by bacteria was crucial for the generation of a cytotoxic effect [19]. Further investigation conducted by Scott *et al* [10] using three-way high-throughput screens verified that bacteria alter the effects of fluoropyrimidines through two distinct pathways. First, bacterial RNA metabolism and vitamins B6 and B9 enable conversion and pro-drug activation. Second, bacteria influence the host metabolic environment, supplying regulatory metabolites that augment 5-FU-induced DNA damage. Concomitant drugs may also affect pro-drug activation. For example, it was also found that metformin inhibits bacterial one-carbon-metabolism, reducing the effect of 5-FU. This highlights the importance of considering all host-microbe-drug interactions when commencing anticancer therapy [10].

Microbial metabolism may cause side effects severe enough to necessitate cessation of chemotherapy. For example, irinotecan-induced mucositis causes severe, dose-limiting diarrhoea in up to 30% of patients [20]. Irinocetan is activated by hydrolysis to form SN-38, an inhibitor of topoisomerase 1. It is later deactivated by hepatic glucuronidation, producing SN-38G, which is excreted into the gut with bile. However, bacterial ß-glucuronidases within the gut lumen can reactivate SN-38G to its active, enterotoxic form, causing mucositis [20]. There are numerous bacterial ß-glucuronidase isoforms, which differ in their substrate pharmacokinetics [21]. Guthrie *et al* [20] recently demonstrated that differential reactivation of SN-38G by healthy volunteers correlated with the presence of specific bacterial ß-glucuronidases and glucuronide membrane transporters [20]. Microbiome characterisation may, therefore, identify patients at risk of irinotecan-induced mucositis while manipulation of the microbiota may provide novel therapeutic options. Ciprofloxacin and low doses of amoxapine have been shown to be effective in the suppression of bacterial ß-glucuronidase activity and mucositis [22, 23]. Furthermore, bacterial ß-glucuronidases possess unique motifs relative to their human counterparts, which pave the way for the development of bacterial-specific inhibitors [22–24].

Inadequate understanding of gut microbiota metabolism can have fatal consequences. This was observed in Japan, when co-administration of the antiviral sorivudine and 5-FU led to toxic circulating levels of 5-FU, causing 16 deaths over a 40 day period [25]. This was caused by inactivation of hepatic dihydropyramidine dehydrogenase (which deactivates 5-FU) by a sorivudine metabolite, BVU [(E)-5-(2-boromovinyl) uracil]. Further investigation revealed that the microbiota, rather than host enzymes, is responsible for producing BVU from sorivudine. Indeed, in a rodent model, circulating levels of BVU and could be abrogated by depletion of gut microbiota with antibiotic administration [18, 26].

## Community structure

Bacterial community structure is a key driver of symbiosis between the host and microbiota and the maintenance of intestinal health [27]. Commensal microbiota can prime and modulate host immunity to prevent pathogen overgrowth. Indeed, *Bacteroides* and *Lactobacillus* have been shown to induce host production of protective antimicrobial proteins within intestinal crypts [28]. The importance of microbiota to maintain the structure and function of the gut mucosal barrier is revealed by analysis of germ-free mice, which display thin villi, a reduced capillary network, a reduction in surface area and poor peristalsis [29, 30]. Administration of chemotherapeutics can damage the diversity and health of gut microbiota, disrupting this equilibrium [31]. In a rat model, methotrexate-induced mucositis and reduced villous length were associated with a 13-fold decrease in protective anaerobes and a 296-fold decrease in *Streptococci* [32]. In humans, 1 week of high-dose chemotherapy for nonHodgkin’s lymphoma dramatically reduces the abundance of commensal *Faecalibacterium* with an increase in the prevalence of potentially pathogenic *Escherichia* [33]. Chemotherapeutic-induced dysbiosis reduces colonisation resistance to pathogens and a decrease in the abundance of anaerobic bacteria may reduce the production of butyrate—a short chain fatty acid with antiinflammatory properties and an essential trophic factor for enterocytes. The resultant damage to the intestinal barrier increases the risk of colitis, bacterial translocation and infection [31].

Efforts to preserve community structure could, therefore, protect against chemotherapeutic injury. It has been shown that healthy microbiota interact, via Toll-like receptors, with the innate immune system, regulating inflammation and promoting healing. For example, antibiotic-treated mice have been shown to be more susceptible to methotrexate-induced small bowel injury, which can be salvaged with Toll-like receptor 2 ligand administration [34]. An analysis of the intestinal microbiome in patients taking anti-CTLA-4 therapy revealed that abundance of the *Bacteriodetes* phylum was associated with resistance to colitis. Administration of *Bacteriodales* has subsequently been shown to reduce CTLA-4 blockade induced colitis in an antibiotic-treated mouse model [4, 35]*.*

## Translation toward clinical practice

As our understanding of pharmacomicrobiomics increases, intestinal microbiome analysis holds the potential to predict patient response to chemotherapy prior to treatment and to personalise therapy. A key example of translation from basic research to clinical application follows an investigation into PD-1 inhibitor immunotherapy, which is highly effective in only a small subset of patients. Following the murine research conducted by Zitvogel *et al* [39] and Sivan *et al* [16] (described earlier), three clinical studies have recently emerged [4, 16, 36–38]. Zitvogel *et al* [39] found that abnormal gut microbiome composition was associated with failure to respond to anti-PD-1 therapy, while faecal microbial transplantation from responding patients to a germ-free murine model was shown to ameliorate chemotherapeutic response. Metagenomic analysis revealed an abundance of *Akkermansia muciniphila* associated positively with clinical response and oral supplementation of the bacterium in germ-free mice restored the response to PD-1 blockade [36]. Gopalakrishnan *et al* [38] examined the microbiomes of melanoma patients undergoing PD-1 immunotherapy. Significantly higher alpha diversity and relative abundance of *Ruminococcaceae* family were observed in responding patients. Immune profiling of patients revealed enhanced systemic and antitumour immunity, which could be transferred to germ-free mice using faecal transplantation [38]. Finally, Gajewski *et al* [37] examined baseline stool samples from metastatic melanoma patients. *Bifidobacterium longum*, *Collinsella aerofaciens* and *Enterococcus faecium* were found to be more abundant in responding patients and faecal microbial transplantation from human responders into germ-free mice restored the antitumour effect of PD-1 blockade in the recipient mice [37].

Although different bacteria were identified, this research holds promise for optimisation of chemotherapeutic efficacy, either through intelligent drug selection or manipulation of microbiota. Various measures may be employed prior to, during and after drug administration. These include selective antibiotic therapy (targeting specific bacterial species), probiotic therapy (administration of living micro-organisms), pre-biotic therapy (compounds administered to encourage the functions of certain microbiota) and post-biotic therapy (use of nonviable microbial products to exert immunological or biological activity) [39]. Use of both antibiotic therapies and probiotic VSL#3 administration has been shown to reduce the incidence of irinocetan-induced mucositis in rodent models [23, 40]. Various studies on probiotic administration during chemotherapy have also suggested that they may reduce the incidence of systemic infection [41]. Ecological manipulation with faecal microbiota transplantation may also hold therapeutic potential [42].

As microbiota may have deleterious as well as advantageous effects, the perfect balance of microbial manipulation may not be possible. For example, while the microbiota may enhance chemotherapeutic response to oxaliplatin [8], they may also engender chemotherapy-induced mechanical hyperalgesia [43]. Shen *et al* [43] demonstrated that the incidence of oxaliplatin-induced mechanical hyperalgesia was lower amongst germ-free mice*.* This protective effect was lost following gut microbial refaunation. As mechanical hyperalgesia affects more than 30% of patients, and can be severe enough to prevent therapeutic dose administration, a compromise may need to be made in microbial manipulation between enhancement of chemotherapeutic effect and reduction of toxicity [43].

Dysbiosis of the gut microbiota following chemotherapeutic treatment may also be used to predict prognosis. Taur *et al* [12] divided patients into low-, intermediate- or high-diversity cohorts based on faecal 16S rRNA analysis shortly after allogenic haematopoietic stem cell transplantation (allo-HSCT). Low microbial diversity was associated with a 31% reduction in 3-year survival compared to high diversity, independent of known predictors and co-morbidities [12]. Furthermore, a study of 857 cases of allo-HSCT revealed that broad-spectrum antibiotic administration during treatment was associated with increased graft-versus-host disease (GVHD)-related mortality at 5 years. The analysis in mice demonstrated that antibiotic administration resulted in an increase in *Akkermansia mucinophilia* and associated loss of the protective mucous lining of the colon, impairment of the intestinal barrier and increased incidence of GVHD [44]. Early studies of faecal microbiota transplantation to tackle steroid-resistant GVHD in four patients have shown promising results [45, 46].

## Novel chemotherapeutics

Innovative possibilities for the genetic engineering of bacteria for delivery of chemotherapy, or as vectors for genetic therapy, are emerging*.* In a study by Pillai *et al* [47]*, E.coli* was genetically modified to enable excretion of a tumour suppressor, human bone morphogenic protein-2 (BMP-2). When exposed to an* in vitro* model of colon cancer, the bacterium induced apoptosis of the adenocarcinoma cell line. Din *et al* [48] used the quorum sensing feedback loops present in *E.coli* to cause the bacterium to lyse synchronously and release Haemolysin E (an antitumour toxin). This created a pulsatile delivery system with simultaneous control of microbial population density. When tested *in vivo* on a mouse model of hepatic colorectal metastases, oral administration of the bacterium, in combination with 5-FU, led to a 50% increase in mean survival time [48].

## Conclusion

During cancer therapy, the gut microbiota is a dynamic structure, influenced by complex interactions between multiple factors, such as host immunity, chemotherapeutics, concomitant medications, environment and diet [6]. The ability of gut microbiota to augment the chemotherapeutic response has been clearly shown in animal models [8, 9, 13, 14, 36–38]. Furthermore, these results have been successfully correlated with clinical research. Specific microbials associated with progression-free survival have been identified amongst patient cohorts and transfer of chemotherapeutic response has been achieved with administration of human faecal samples to a murine model [36–38]. These findings have tremendous clinical potential, particularly in the field of novel immunotherapeutics (such anti-PD-L1), which at present are highly effective for only a small subset of patients [36–38]. However, gut microbes are not always beneficial. An increased understanding of gut microbial metabolism has revealed contributions to drug toxicity such as mucositis and neuropathy that can even prove fatal [22, 23, 25, 26, 43]. The delicate equilibrium of bacterial community structure and host-microbiota interactions is also disrupted by chemotherapeutic administration, which promotes inflammation and mucosal destruction, and is associated with adverse outcome [31–34].

The functional diversity of the microbiota has the capacity for both clinical benefit and harm. Obtaining a comprehensive understanding of the role of gut microbiota and their role in chemotherapy will require a dedicated and innovative systems medicine approach. Manipulation of the gut microbiota to achieve a perfect balance of chemotherapeutic efficacy and reduction of side effects presents a significant challenge [43]. However, with the potential of pharmacomicrobiomics to deliver a new era of cancer treatment, improving efficacy and tolerability, it is surely worth the effort.

## Conflicts of interest

The authors declare no conflicts of interest.
